# Daily Social Media Use, Social Ties, and Emotional Well-Being in Later Life

**DOI:** 10.1093/geroni/igab046.1169

**Published:** 2021-12-17

**Authors:** Yijung Kim, Karen Fingerman

**Affiliations:** The University of Texas at Austin, Austin, Texas, United States

## Abstract

Research has seldom explored older adults’ daily social media use and its interface with ‘offline’ social ties. Using data from the Daily Experiences and Well-being Study (N = 310; Mage = 73.96), we investigated whether more daily social media use was associated with the same-day negative or positive mood in later life, and how these associations varied with older adults’ daily social encounters and social network structure. More daily social media use was associated with less same-day negative mood. Additionally, more daily social media use was associated with less negative mood on days with more in-person encounters, compared to the days with fewer in-person encounters. More daily social media use was also associated with more positive mood for individuals with a relatively small social network, but not for their counterparts. Post-hoc analyses supported a compensatory function of social media for those older adults lacking social connections in their daily lives.

